# Targeting *Toll-like* Receptor 4/Nuclear Factor-κB and *Nrf2*/Heme Oxygenase-1 Crosstalk via Trimetazidine Alleviates Lipopolysaccharide-Induced Depressive-like Behaviors in Mice

**DOI:** 10.1007/s11481-024-10149-3

**Published:** 2024-09-23

**Authors:** Sarah S. Mohamed, Nora O. Abdel Rasheed, Weam W. Ibrahim, Nesma A. Shiha

**Affiliations:** Department of Pharmacology and Toxicology, Faculty of Pharmacy, Cairo University, Kasr El Aini St, Cairo, Egypt

**Keywords:** Lipopolysaccharide, Trimetazidine, Depression, Mice, NF-κB, Nrf2

## Abstract

Depression is a global psychiatric illness that imposes a substantial economic burden. Unfortunately, traditional antidepressants induce many side effects which limit patient compliance thus, exploring alternative therapies with fewer adverse effects became urgent. This study aimed to investigate the effect of trimetazidine (TMZ); a well-known anti-ischemic drug in lipopolysaccharide (LPS) mouse model of depression focusing on its ability to regulate toll-like receptor 4 (TLR4)/nuclear factor-κB (NF-κB) as well as nuclear factor erythroid 2 related factor 2 (Nrf2)/ heme oxygenase-1 (HO-1) signaling pathways. Male Swiss albino mice were injected with LPS (500 µg/kg, i.p) every other day alone or parallel with oral doses of either escitalopram (Esc) (10 mg/kg/day) or TMZ (20 mg/kg/day) for 14 days. Treatment with TMZ attenuated LPS-induced animals' despair with reduced immobility time inforced swimming test. TMZ also diminished LPS- induced neuro-inflammation via inhibition of TLR4/NF-κB pathway contrary to Nrf2/HO-1 cascade activation with consequent increase in reduced glutathione (GSH) and HO-1 levels whereas the pro-inflammatory cytokines; tumor necrosis factor-α (TNF-α) and interleukin (IL)-1β were evidently reduced. Besides, TMZ replenished brain serotonin levels via serotonin transporter (SERT) inhibition. Thus, TMZ hindered LPS-induced neuro-inflammation, oxidative stress, serotonin deficiency besides its anti-apoptotic effect which was reflected by decreased caspase-3 level. Neuroprotective effects of TMZ were confirmed by the histological photomicrographs which showed prominent neuronal survival. Here we showed that TMZ is an affluent nominee for depression management via targeting TLR4/NF-κB and Nrf2/HO-1 pathways. Future research addressing TMZ-antidepressant activity in humans is mandatory to enroll it as a novel therapeutic strategy for depression.

**Graphical Abstract:**

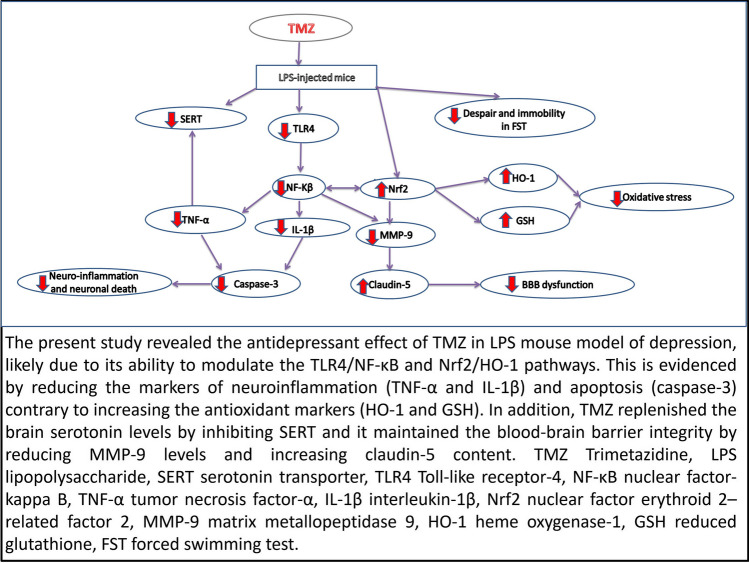

## Introduction

Depression is a common disabling mental disorder that is estimated to affect 5% of adults across the world (World Health Organization 2023). It is characterized by a cluster of symptoms that includes low mood and energy, loss of interest and pleasure, sleeping and eating disturbances, cognitive impairment, and suicidal ideation (Maffioletti et al. 2020; Li et al. 2021). These symptoms are associated with structural and neurochemical changes in mesolimbic and corticolimbic neural circuits which regulate mood and behavior, including the prefrontal cortex (PFC), amygdala, nucleus accumbens, and hippocampus (Afridi and Suk 2021). Decreased gray matter density in the hippocampus, amygdala and PFC has been reported in patients with depression (Yu et al. 2023). Moreover, volumes of the whole brain and hippocampus of depressed patients were found to be lower than those of the control group (Li et al. 2021).

The monoamine hypothesis is a widely embraced theory, which suggests that depression is attributed to dysregulations of monoamine neurotransmitters such as serotonin, norepinephrine, and dopamine (Lee et al. 2010). However, over the years, several other pathophysiological mechanisms have been implicated in the disease pathogenesis including impaired neurogenesis and synaptic plasticity, depleted neurotrophic factors, neuroinflammation, and oxidative stress (Jazvinšćak Jembrek et al. 2023; Fang et al. 2023). Postmortem findings of reduced hippocampal size and volume implicates dysregulated hippocampal neurogenesis in major depressive disorder (MDD) pathogenesis (Fries et al. 2023). This is primarily caused by disturbance in metabolic levels of neurotrophic factors such as brain-derived neurotrophic factor (BDNF) (Kamran et al. 2022). The latter is known to play a critical role in neurogenesis, neuronal survival and differentiation, synaptic plasticity, neuroprotection, learning, memory and mood control (Correia and Vale 2024).

Numerous studies have reported a significant association between neuro-inflammation and depression (Maffioletti et al. 2020; Owen et al. 2001; Raison et al. 2006; Goldsmith et al. 2016). The core features of inflammatory responses such as increased pro-inflammatory cytokine levels and abnormal changes in oxidative stress parameters including malondialdehyde (MDA), superoxide dismutase (SOD), and glutathione-S-transferase (GST) have been reported in blood and brain samples of patients with MDD (Zhuo et al. 2022**).** Neuroinflammation is implicated in the pathophysiology of depression by increasing the proinflammatory cytokines, activating the hypothalamic–pituitary–adrenal (HPA) axis, interfering with the synthesis and metabolism of monoamines, increasing the neuronal apoptosis, and impairing the neurogenesis and neuroplasticity (Evrensel et al. 2020; Kouba et al. 2024).

In accordance, several studies have demonstrated that administration of the bacterial endotoxin lipopolysaccharide (LPS) can cause depressive symptoms in rodents (Liu et al. 2016; Stupp et al. 2018; Zhang et al. 2019). LPS triggers immune responses by acting on toll-like receptor 4 (TLR4), a vital pattern recognition receptor (PRR) (Ciesielska et al. 2021; Nonoguchi et al. 2022). Upon binding to TLR4, LPS can activate nuclear factor-κB (NF-κB) which induces the production of inflammatory cytokines such as interleukin (IL)-1β, IL-6, and tumor necrosis factor-α (TNF-α), eventually promoting oxidative stress and decreasing the expression of BDNF (Wei et al. 2022; Taniguti et al. 2018).

Oxidative stress is a major contributor to the pathogenesis of various neurological diseases such as Alzheimer’s disease, Parkinson’s disease, multiple sclerosis, and depression (Correia et al. 2023; Pizzino et al. 2017). It results from the imbalance between the production of free radicals, particularly reactive oxygen species (ROS) and antioxidant defense systems (Correia et al. 2023). The increased ROS levels are known to damage biological macromolecules such as nucleic acids, proteins, and lipids resulting in cellular damage (Bian et al. 2020; Arioz et al. 2019). Nuclear factor erythroid 2 related factor 2 (Nrf2) is a key transcription factor that regulates cellular antioxidant responses (Bian et al. 2020). It activates antioxidant response element (ARE)-dependent gene expression of various antioxidant and cytoprotective proteins such as SOD-1, glutathione peroxidase (GPx), GST, and heme oxygenase-1 (HO-1) (Abou El-Ezz et al. 2018). The latter is an enzyme that possesses anti-inflammatory and antioxidant activities (Bian et al. 2020). Nrf2 inhibition is reportedly involved in the development of MDD. Indeed, decreased prefrontal cortex expression of Nrf2 has been documented in both MDD patients and experimental models of depression. On the other hand, compounds activating Nrf2 have shown considerable antidepressant effects in preclinical studies, suggesting that targeting Nrf2 could be a promising strategy to combat depression (Zuo et al. 2022).

Blood–brain barrier (BBB) is essential for maintenance of the central nervous system (CNS) homeostasis (Huang et al. 2021). It is formed by specialized endothelial cells that line the cerebral microvasculature and regulates the passage of molecules and cells between the blood and the brain (Gastfriend et al. 2018; Daneman and Prat 2015). Therefore, the BBB protects the brain from toxins, pathogens, inflammation, injury, and diseases (Daneman and Prat 2015). The integrity of the BBB is maintained mainly by tight junction proteins such as claudin-5, whose loss has been implicated in loosening of the BBB and increased permeability (Zhou et al. 2014; Menard et al. 2017). BBB disruption has been involved in the pathogenesis of different CNS diseases such as depression, epilepsy and schizophrenia (Ibrahim et al. 2022). Moreover, peripheral inflammation including LPS-induced inflammation is reported to disrupt the BBB via various pathways (Huang et al. 2021). Indeed, LPS reportedly reduced tight junction proteins such as claudin-5 and occludin in young and old mice thus affecting BBB integrity (Zhou et al. 2014; Wang et al. 2017**).**

Trimetazidine (TMZ) is a cytoprotective anti-ischemic drug widely used in the treatment of coronary artery disease (Dézsi 2016). It is a selective inhibitor of long‐chain 3‐ketoacyl coenzyme A thiolase; an enzyme which catalyzes the final step in β-oxidation, thus shifting energy production from free fatty acids oxidation towards glucose oxidation leading to suppression of oxygen demands and enhancement of myocardial efficiency (Abdel-Salam et al. 2011). Cardioprotective effects of TMZ have also been attributed to its reported antioxidant and anti-inflammatory effects (Zou et al. 2017). Besides its cardioprotective actions, the neuroprotective properties of TMZ have been getting more attention over the past years. TMZ has exhibited neuroprotective effects against focal cerebral ischaemia–reperfusion injury in rats (Dhote and Balaraman 2008) and anxiolytic effects in animal models of increased anxiety, owing to its antioxidant activity (Kolik et al. 2017). It has also been reported that treatment with TMZ ameliorated cognitive impairment in diabetic epileptic rats by hampering the inflammatory process (Mohamed et al. 2020). Importantly, TMZ was recently identified as a potential drug that may be repurposed to treat bipolar depression using a combination of transcriptomics, drug screening and in vitro as well as in vivo mechanistic studies conducted by Bortolasci et al. (2023).

Accordingly, the current study sought to investigate the potential benefit of TMZ in attenuating LPS-induced depressive-like behaviors, neuro-inflammation, apoptosis, oxidative stress and BBB dysfunction in mice. Special emphasis was set on the possible involvement of the TLR4/NF-κB and Nrf2/HO-1 pathways in the protective effects of TMZ.

## Materials and Methods

### Animals

Adult male Swiss albino mice weighing 22–25 g were used in the current study. They were obtained from the animal facility of the National Research Centre, Giza, Egypt and allowed to acclimate for 1 week before starting the experiment. Animals were maintained under temperature- and humidity-controlled conditions on a 12/12 light–dark cycle and provided access to food and water ad libitum. The experimental procedures were performed in accordance with the Guide for the Care and Use of Laboratory Animals published by the US National Institutes of Health (NIH Publication No. 85–23, revised 2011) and were approved by the Ethics Committee for Animal Experimentation of Faculty of Pharmacy, Cairo University (PT3522). Efforts were made to reduce animal suffering and decrease the number of animals used.

### Materials

Lipopolysaccharide (LPS) (Escherichia coli, serotype 0127: B8) was purchased from Sigma-Aldrich, USA. Trimetazidine dihydrochloride (TMZ) and escitalopram (Esc) were acquired from Servier and Multi-Apex Pharmaceutical Company, Egypt, respectively. They were all freshly prepared using physiological saline before use.

### Induction of Depressive-like Behavior Model in Mice

Induction of a depressive-like behavior model was achieved by intraperitoneal injection of LPS at a dose of 500 µg/kg, every other day for 14 days, for a total of 7 injections. This study was performed in accordance with the previous investigations (Yang et al. 2018; Jiang et al. 2017; Abdel Rasheed et al. 2023; Gong et al. 2019), which showed that repeated peripheral LPS administration effectively provoked depressive-like behaviors and neuroinflammation in rodents.

### Experimental Design

**Fig. 1:**
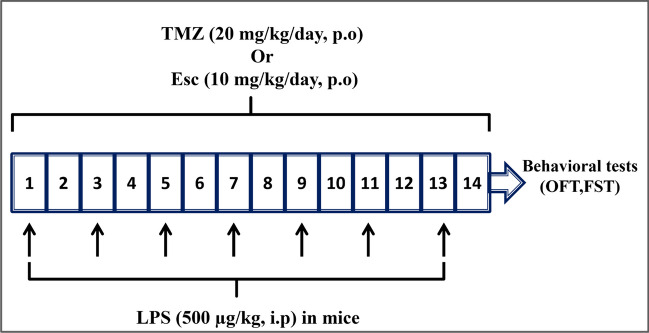
Representation of the experimental design. *LPS* lipopolysaccharide, *TMZ* Trimetazidine, *Esc* escitalopram, *OFT* open field test, *FST* forced swimming test

Mice were randomly divided into 5 groups (n = 15/group) as follows; control, TMZ, LPS, LPS + Esc, and LPS + TMZ groups (Fig. 1**)**. Mice were treated orally with either Esc (10 mg/kg/day) (Kurhe and Mahesh 2016; Ibrahim et al. 2019) or TMZ (20 mg/kg/day) (Kolik et al. 2017; Jain et al. 2011; Engin et al. 2022) for 14 days covering the LPS administration period. Following treatments, mice were behaviorally tested using open field test (OFT) and forced swimming test (FST). After performing the behavioral tests, animals were euthanized by decapitation under anesthesia, then the whole brains were carefully collected and washed with ice-cold saline. Brains of three mice from each group were kept in 10% formalin for histopathological examination of the hippocampal region and for determination of mean intact neurons count. The hippocampi of the remaining mice were dissected out, flash frozen in liquid nitrogen and stored at − 80°C for further biochemical investigations. Six of these hippocampi were then homogenized in cold phosphate-buffered saline to prepare a 10% homogenate to be used for enzyme-linked immunosorbent assay (ELISA) tests while the remaining six were used for real-time PCR analysis.

### Behavioral Assessment

#### Open Field Test

Locomotor function of the mice was evaluated using the OFT. The test was performed in a wooden box (80 × 80 × 40 cm) with red walls and a black floor. The floor of the box was divided into 16 equal squares by white lines. Each mouse was placed in the middle of the open field and left to explore for 3 min. The arena was cleaned carefully using 20% alcohol between each mouse to get rid of any remnant odors. The animal's locomotor function was assessed by recording the frequencies of ambulation and rearing which are the number of squares crossed and rearing, respectively (Ibrahim et al. 2022; Abdel Rasheed et al. 2018).

#### Forced Swimming Test

Forced swimming test was performed to assess the depressive-like behaviors in rodents (Liu et al. 2016). Mice were individually placed in a transparent cylinder (height: 40 cm, diameter: 30 cm) containing about 35 cm of water (25 ± 1°C) for 5 min. The total amount of time each animal remained immobile was recorded as the immobility time. The animal was considered immobile when floating motionless or making only small movements needed to keep its head above the water level (Zhang et al. 2014; Chai et al. 2019**).**

### Histopathological Examination

Brain samples were fixed in neutral buffered formalin (10%) for 72 h. Samples were then processed in serial grades of ethanol and cleared in xylene. Afterwards, they were infiltrated and embedded into Paraplast tissue embedding media. A rotatory microtome was used to cut 5 μm-thick serial sagittal brain sections for demonstration of hippocampal regions in different samples and mounted on glass slides. Sections were then stained with hematoxylin and eosin (H&E) as a general staining method for tissue examination. Standard procedures for sample fixation and staining were carried out according to Culling (2013). Additionally, sections were stained with toluidine blue and six randomly selected non-overlapping fields from the CA3 hippocampal region of each sample were assessed to determine mean intact neurons count (Gad et al. 2022). All light microscopic examination and data were obtained using a full HD microscopic imaging system and Leica application module for tissue section analysis (Leica Microsystems GmbH, Germany).

### Biochemical Assays

#### Enzyme-Linked Immunosorbent Assay

Hippocampal NF-κB and matrix metalloproteinase-9 (MMP-9) contents were measured using mouse ELISA kits supplied by LifeSpan Biosciences (USA) and Elabscience (China), respectively. Moreover, mouse MyBioSource ELISA kits (USA) were used for the assessment of TNF-α, Nrf2, HO-1, GSH, and claudin-5 contents in the hippocampus, while IL-1β and caspase 3 contents were estimated using R&D systems mouse ELISA kits (USA). The procedures were performed following the protocols provided by the manufacturers. Protein content was determined using the method described by Lowry et al. (1951).

#### Quantitative Real-Time PCR Analysis

Total RNA was extracted from the mice hippocampi using SV total RNA isolation system (Promega, USA) and purity of the obtained RNA was detected spectrophotometrically at 260/280 nm. Using the Reverse Transcription System (Promega, USA), the extracted RNA was reverse transcribed into complementary DNA. To evaluate gene expression of TLR4 and serotonin transporter (SERT), quantitative real-time PCR was performed using the SYBR Green JumpStart Taq ReadyMix (Sigma-Aldrich, USA) as per the manufacturer's instructions. The used primers' sequences are illustrated in Table 1. The relative expression of the target genes was estimated using the 2^−∆∆CT^ formula (Livak and Schmittgen 2001) with β-actin used as a housekeeping gene.

**Table 1:** Sequences of the primers used for quantitative real time-PCR analysis

Gene
**Primer sequence**
TLR4
Forward: 5′-ATGCATGGATCAGAAACTCAGCAA-3′
Reverse: 5′- AAACTTCCTGGGGAAAAACTCTGG-3′
SERT
Forward: 5′-AAGCCCCACCTTGACTCCTCC-3′
Reverse: 5′-CTCCTTCCTCTCCTCACATATCC-3′
β-actin
Forward: 5′-CACTGTCGAGTCGCGTCC-3′
Reverse: 5′-CGCAGCGATATCGTCATCCA-3′

### Statistical Analysis

Data sets that met the requirements for parametric criteria were analyzed using one-way ANOVA followed by Tukey’s multiple comparisons test. Results were presented as mean ± SD. Statistical analysis was performed using GraphPad Prism software, version 9 (GraphPad Software Inc., USA) with the significance level fixed at P < 0.05. For each effect, the F-value (F), the degree of freedom, and the statistical significance (p) were reported, moreover, pairwise comparisons were provided in each corresponding figure.

## Results

The results displayed by TMZ group were presented herein concurrently with the other experimental groups. They were insignificantly different as compared to those of the control group upon histological, behavioral, and biochemical investigations. Thus, comparisons were done as related to the control group.

### Trimetazidine Alleviated LPS-Induced Depressive-like Behavior in Forced Swimming Test whereas it Exerted Null Effect on Locomotor Activity in Open Field Test

**Fig. 2:**
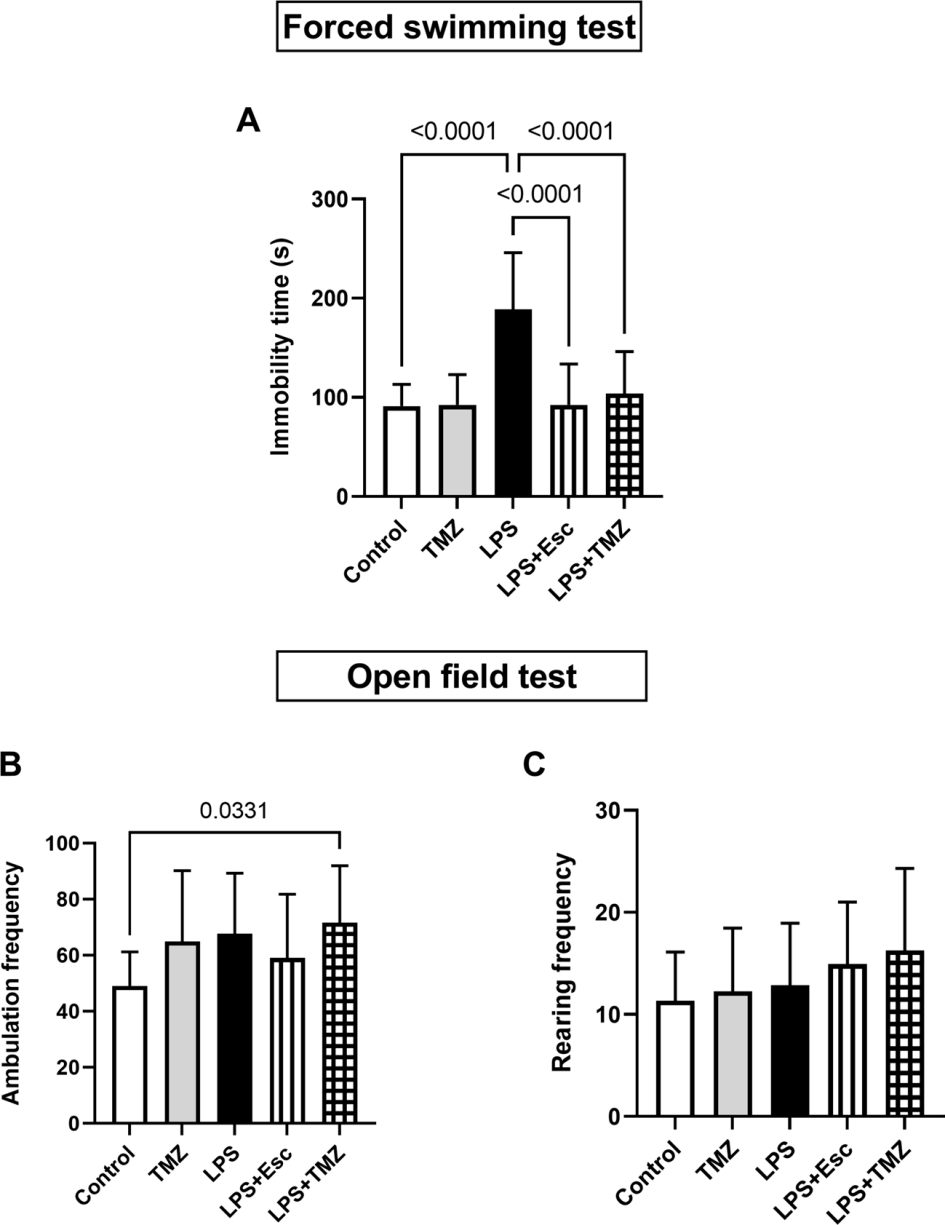
Trimetazidine alleviated LPS-induced behavioral changes in forced swimming and open field tests.** (A)** Immobility time, **(B)** Ambulation frequency, and **(C)** Rearing frequency. Data were expressed as mean ± SD (n = 15) using one-way ANOVA followed by Tukey's post-hoc test, P < 0.05.* LPS* lipopolysaccharide, *TMZ* Trimetazidine, *Esc* escitalopram

LPS injection in mice instigated depressive-like symptoms which were investigated behaviorally using the FST. In comparison with the control group, LPS-injected mice showed a state of despair in FST where they displayed a marked rise in the time of immobility by 2.1-folds (F_(4, 70)_ = 16.33, p < 0.0001). Treatment with either Esc or TMZ ameliorated LPS-induced depressive-like behavior restoring the values of immobility time to the normal range. As compared to the LPS group, Esc- or TMZ-treated mice exhibited reduced immobility duration by 51 and 45%, respectively **(**Fig. 2A**)**.

In the open field test, the ambulation and rearing frequencies were documented to evaluate the impact of LPS, TMZ, or Esc on spontaneous locomotor activity of rats. There was insignificant variation among the experimental groups in either the frequency of ambulation (F_(4, 70)_ = 2.658, p = 0.0398) or rearing (F_(4, 70)_ = 1.528, p = 0.2035). These findings rule out the possibility that the depressive-like anomalies detected herein can be attributed to changes in locomotor function **(**Figs. 2B and C**)**.

### Trimetazidine Alleviated LPS-Induced Hippocampal Histopathological Changes in Mice

**Fig. 3:**
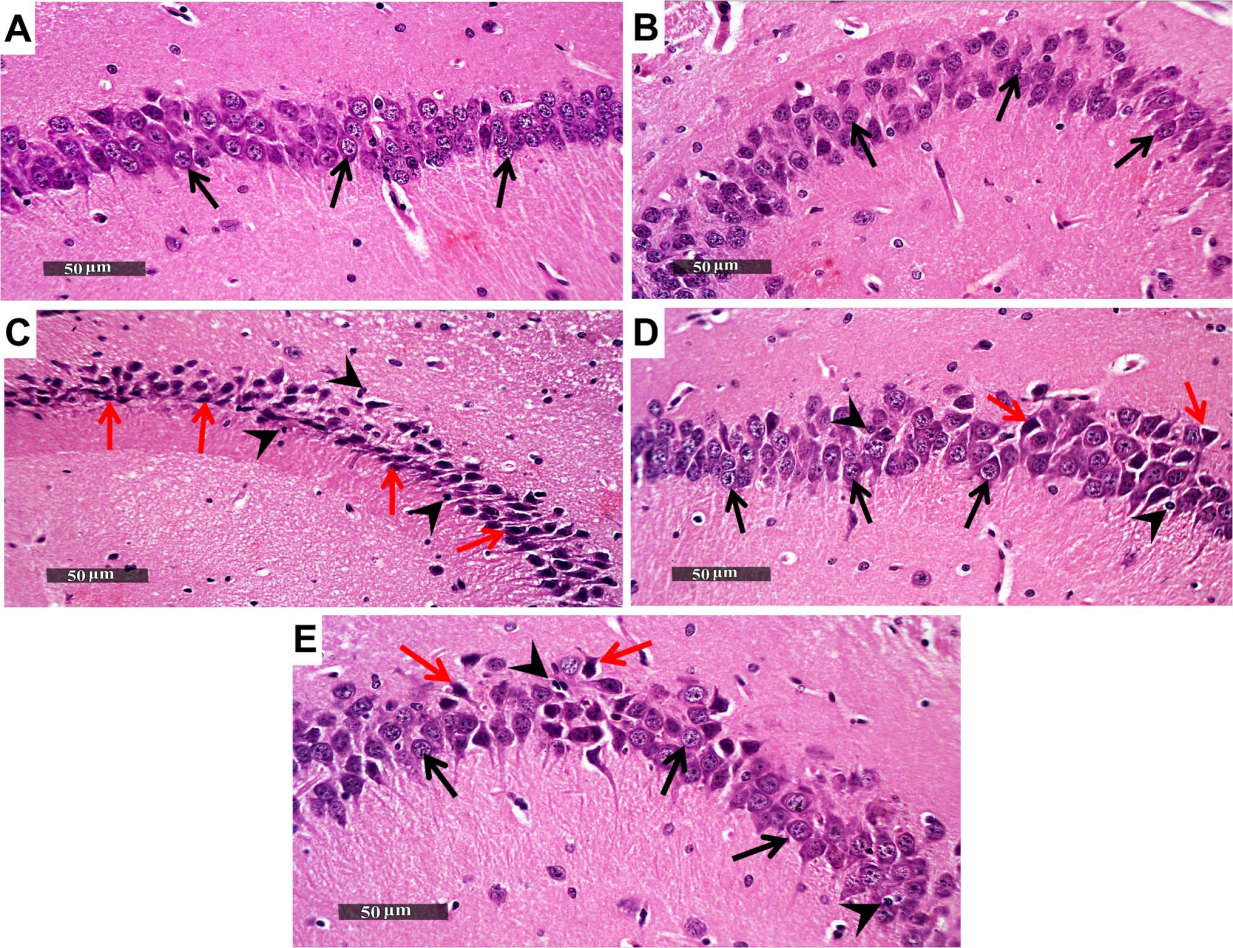
Trimetazidine alleviated LPS-induced hippocampal histopathological changes in mice. Representative H&E photomicrographs of all experimental groups (n = 3); Control, TMZ, LPS, LPS + Esc, and LPS + TMZ groups. Magnifications: × 400. *Black arrows* indicate intact neurons while *red arrows* represent degenerated ones. *Arrowheads* show reactive glial cells infiltrate.* LPS* lipopolysaccharide, *TMZ* Trimetazidine, *Esc* escitalopram

**Fig. 4:**
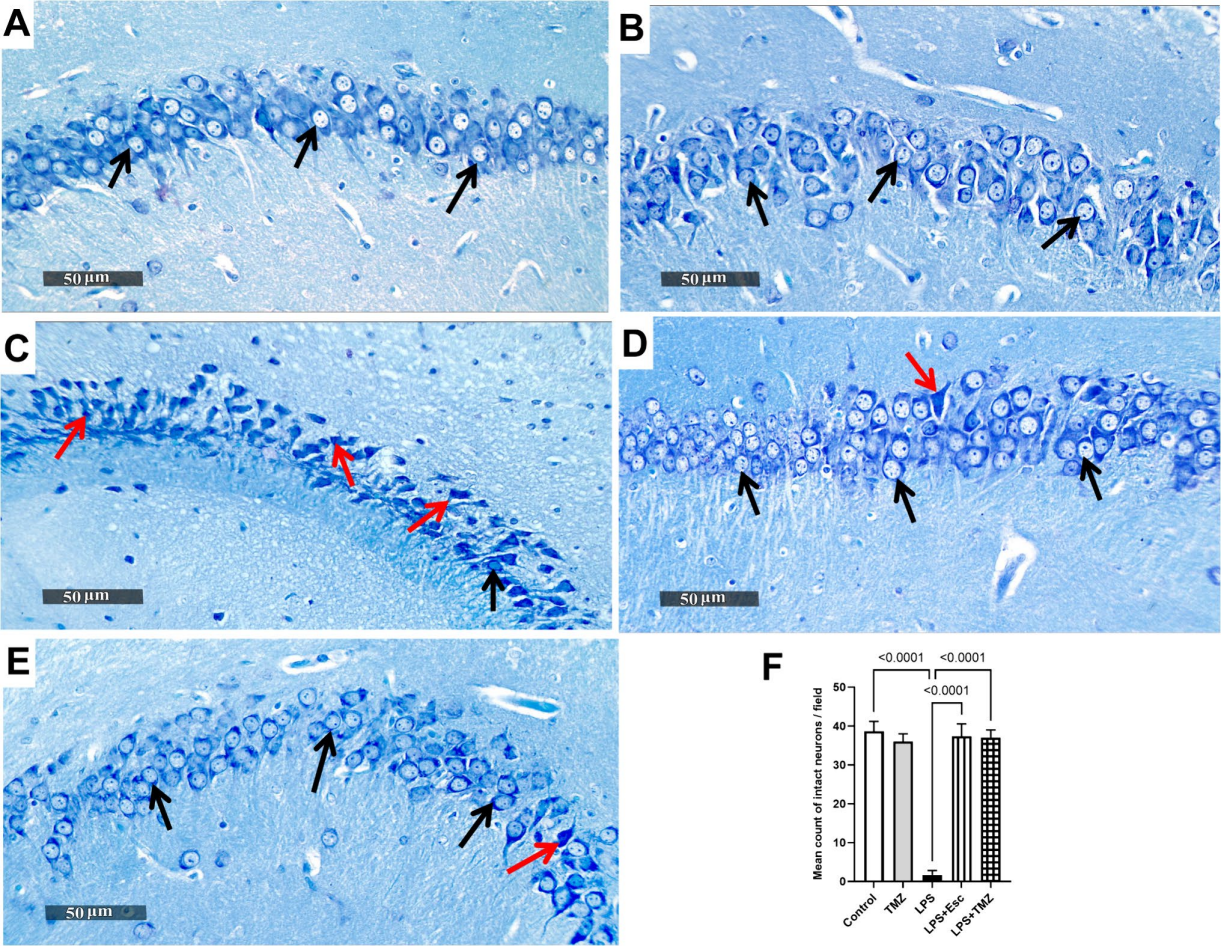
Trimetazidine enhanced hippocampal neuronal survival in LPS-injected mice. Illustrative toluidine blue-stained photomicrographs of all experimental groups; Control, TMZ, LPS, LPS + Esc, and LPS + TMZ groups. Magnifications: × 400. *Black arrows* represent intact neurons while *red arrows* show degenerated ones. A bar chart showing the mean count of intact neurons in each group; where each bar with a vertical line illustrating the mean ± SD (n = 6), using one-way ANOVA followed by Tukey's post-hoc test, P < 0.05.* LPS* lipopolysaccharide, *TMZ* Trimetazidine, *Esc* escitalopram

Representative photomicrographs of the H&E-stained samples of different groups were microscopically examined, in addition to using toluidine-blue stain for determining the intact neurons count in CA3 hippocampal area. The control group showed normal histological appearance of the hippocampal layers along with many records of apparent intact pyramidal neurons having well recognized nuclear and cytoplasmic details (black arrows), in addition to an intact brain matrix with the absence of cellular infiltrates. Similarly, the hippocampal sections of the TMZ group presented comparable histopathological appearance with that of the control group without recording any abnormal changes. In contrast, the histological examination of LPS group samples showed obvious dispersed neuronal degeneration as indicated by ample of damaged or necrotic neurons having pyknotic perikarya missing its subcellular details, along with mild perineuronal edema (red arrows) in contrast to reduced scattered records of intact cells (black arrows). Additionally, LPS group samples demonstrated a remarkable increase in reactive microglial cell infiltrates (arrowhead). Upon examining the toluidine blue-stained sections, LPS group presented a noticeable decline in the intact neurons mean count by 96% as compared to the control group (F_(4, 25)_ = 382.8, p < 0.0001). Notably, treatment with either Esc or TMZ afforded equivalent neuroprotective efficacy against LPS-induced histopathological changes. The brain samples of Esc-or TMZ-treated mice exhibited few sporadic records of damaged neurons (red arrow) and higher records of apparent intact neurons (black arrow) with intact intercellular brain matrix and minimal infiltrates (arrowhead), as compared to the LPS group **(**Figs. 3 and 4**)**.

### Trimetazidine Mitigated LPS-Induced Activation of Hippocampal TLR4/NF-κB Pathway

**Fig. 5:**
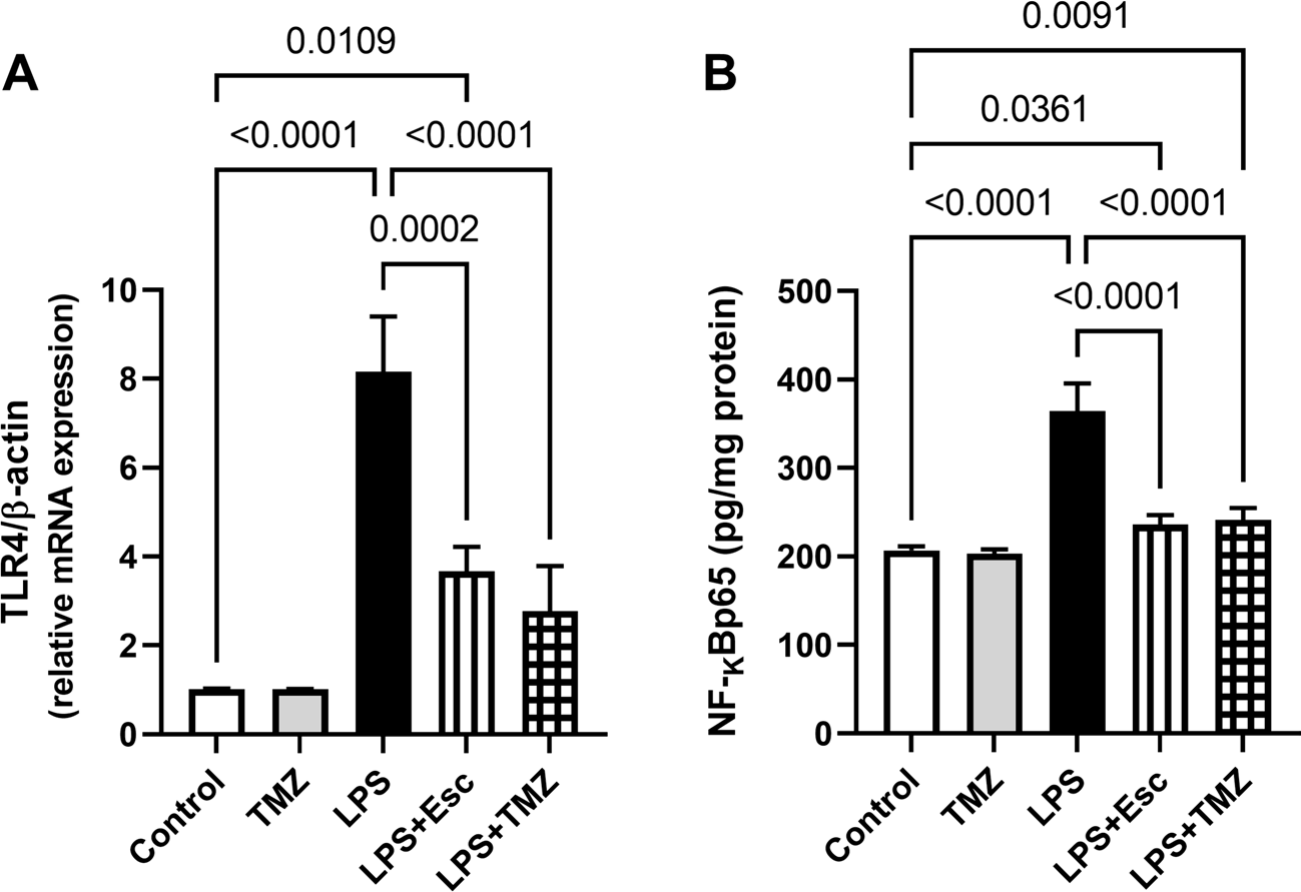
Trimetazidine mitigated LPS-induced alterations in the hippocampal **(A)** TLR4 gene expression and **(B)** NF-κBp65 content. Data were expressed as mean ± SD (n = 6), using one-way ANOVA followed by Tukey's post-hoc test, P < 0.05.* LPS* lipopolysaccharide, *TMZ* Trimetazidine, *Esc* escitalopram.* TLR4* Toll-like receptor-4,* NF-κB* nuclear factor-kappa B

The LPS injection led to a profound activation of TLR4/NF-κB pathway as evidenced by producing a considerable increase in the hippocampal gene expression of TLR4 by 8-folds, with subsequent increment in the hippocampal content of NF-κBp65 by 1.8-folds, as compared to the control group (for TLR4: F_(4, 25)_ = 112.8, p < 0.0001; for NF-κBp65: F_(4, 25)_ = 98.69, p < 0.0001). Upon treating the LPS-exposed mice with TMZ, a marked drop in TLR4 gene level as well as NF-κBp65 content by 66 and 34%, respectively, was detected producing an equivalent effect with that displayed by the LPS + Esc group **(**Fig. 5**).**

### Trimetazidine Reduced LPS-Induced Neuro-Inflammation, Apoptosis and Oxidative Stress

**Fig. 6:**
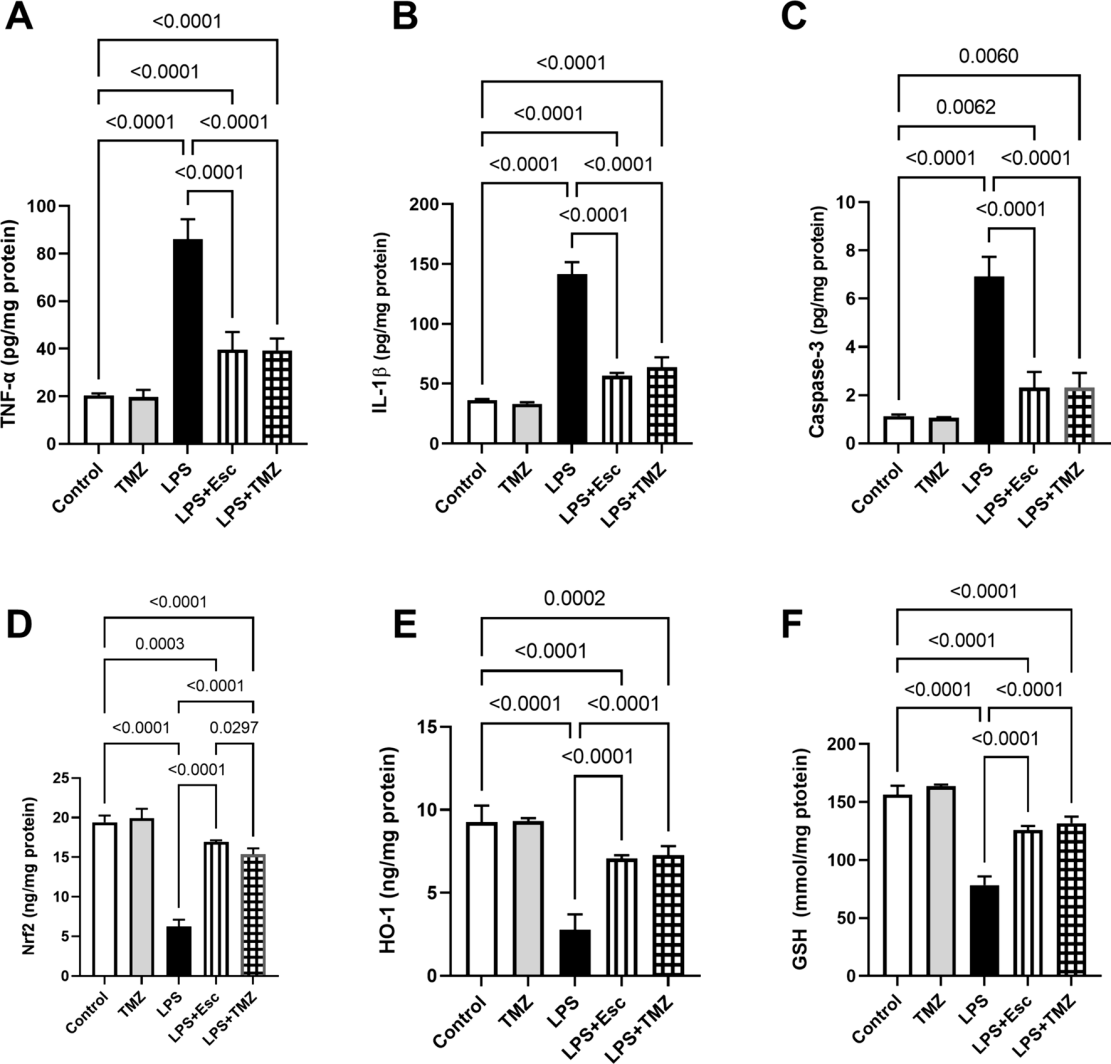
Trimetazidine reduced LPS-induced alterations in the hippocampal contents of **(A)** TNF-α, **(B)** IL-1β, **(C)** Caspase-3, **(D)** Nrf2, **(E)** HO-1, and **(F)** GSH. Data were expressed as mean ± SD (n = 6), using one-way ANOVA followed by Tukey's post-hoc test, P < 0.05.* LPS* lipopolysaccharide, *TMZ* Trimetazidine, *Esc* escitalopram, *TNF-α* tumor necrosis factor-α,* IL-1β* interleukin-1β, *Nrf2* nuclear factor erythroid 2–related factor 2, *HO-1* heme oxygenase-1, *GSH* reduced glutathione

The LPS depression model was accompanied by aggravated responses of neuro-inflammation, apoptosis, and oxidative stress. In comparison to the control group, LPS-injected mice showed a profound rise in the hippocampal contents of inflammatory markers, TNF-α (4.2-folds) and IL-1β (3.9-folds), along with a significant increment in the proapoptotic caspase-3 concentration by 6.1-folds (F_(4, 25)_ = 137.0, 318.4, and 119.9, respectively, p < 0.0001). On the contrary, they showed a considerable decrease in the antioxidant variables; namely Nrf2, HO-1, and GSH levels by 68, 70, and 50%, respectively, in comparison to their control counterparts (F_(4, 25)_ = 256.3, 94.84, and 204.8, respectively, p < 0.0001). Treatment of LPS-injected mice with TMZ exerted neuroprotective effects leading to about 54% depression in TNF-α and IL-1β concentration along with marked decline in caspase-3 content (66%). Additionally, TMZ mitigated LPS-induced oxidative stress producing significant rise in Nrf2 (2.5-folds), HO-1 (2.6-folds), and GSH levels (1.7-folds). Such effects of TMZ on the previously mentioned markers were almost comparable with those displayed by Esc treatment except for Nrf2 where Esc outweighed the TMZ effect producing 10% increase in Nrf2 content as compared to the TMZ-treated group** (**Fig. 6**)**.

### Trimetazidine Diminished LPS-Induced Surge in Hippocampal SERT Expression

**Fig. 7:**
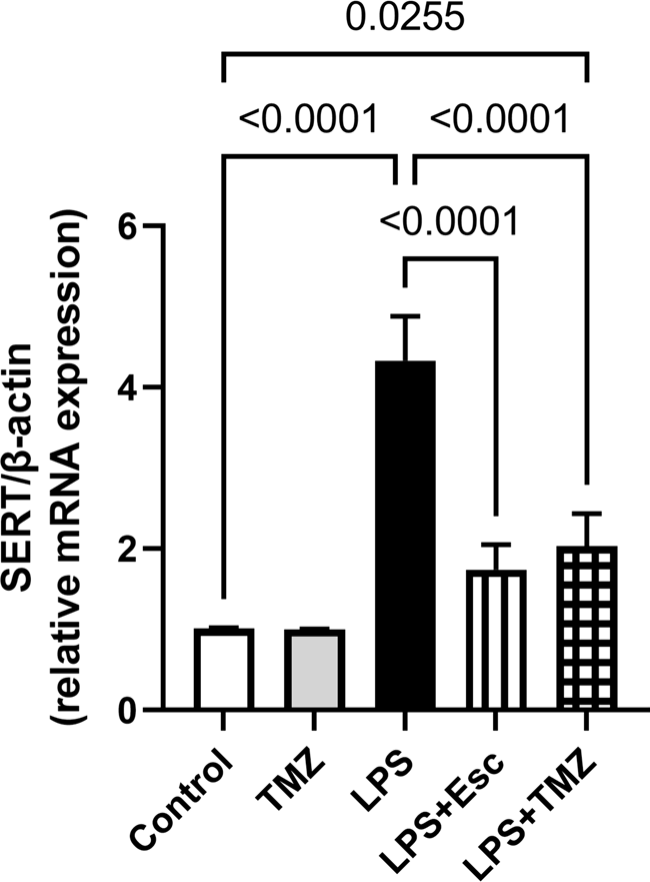
Trimetazidine diminished LPS-induced changes in the hippocampal SERT gene expression. Data were expressed as mean ± SD (n = 6) using one-way ANOVA followed by Tukey's post-hoc test, P < 0.05.* LPS* lipopolysaccharide, *TMZ* Trimetazidine, *Esc* escitalopram, SERT serotonin transporter

The administration of LPS in mice notably augmented the hippocampal mRNA expression of SERT by 4.3-folds, in comparison to the control group (F_(4, 25)_ = 124, p < 0.0001). This effect was inhibited by the TMZ administration where LPS + TMZ group mice showed a noticeable reduction in SERT gene level by 53%, as compared to the LPS group. Likewise, Esc administration obviously decreased the SERT gene level by 60%, as compared to the LPS group. Results afforded by the LPS + Esc and LPS + TMZ groups on SERT expression in the hippocampi were almost comparable where no substantial change was revealed between the two groups **(**Fig. 7**)**.

### Trimetazidine Attenuated LPS-Induced Blood-*Hippocampus* Barrier Dysregulation

**Fig. 8:**
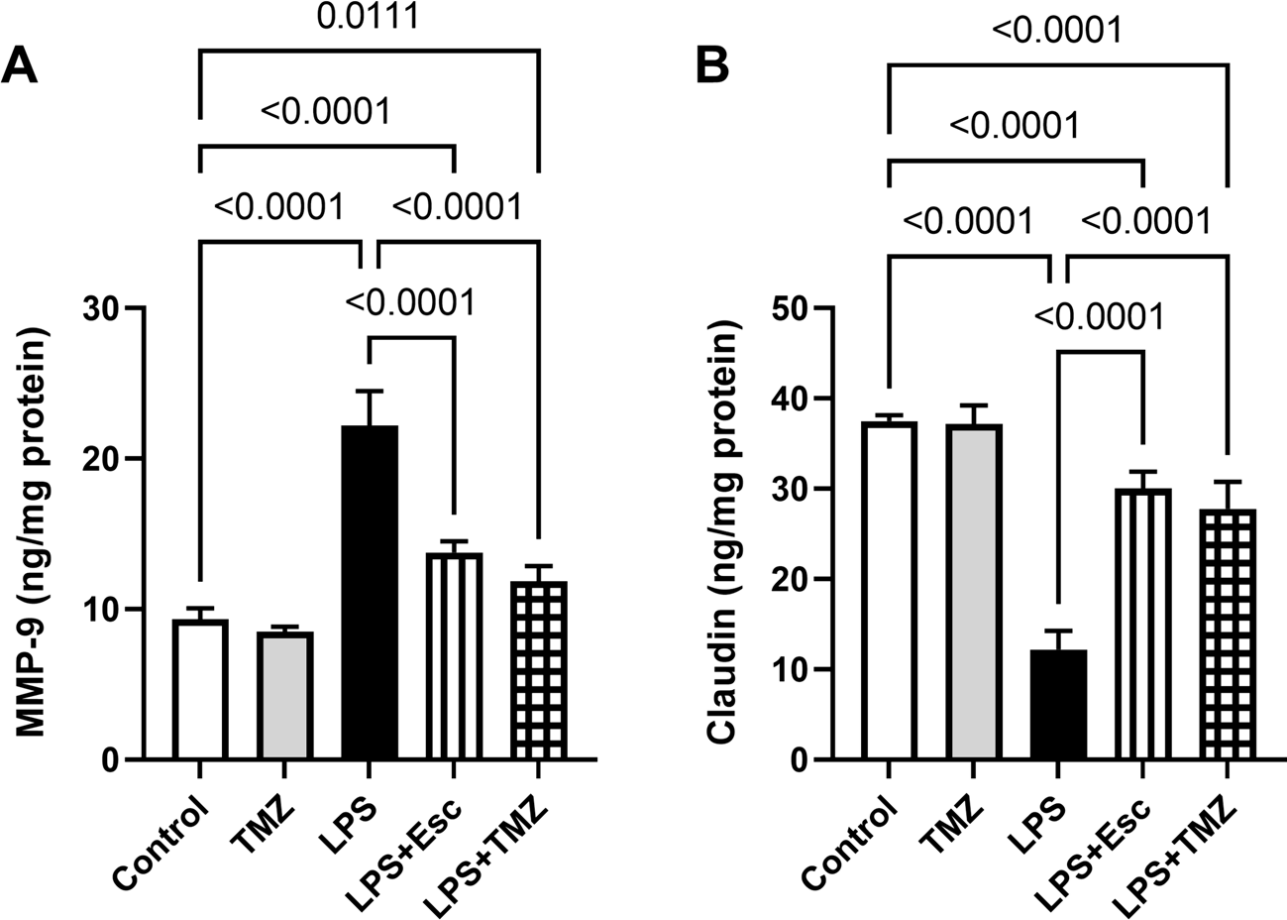
Trimetazidine attenuated LPS-induced changes in the hippocampal contents of (A) MMP-9 and (B) Claudin-5. Data were expressed as mean ± SD (n = 6) using one-way ANOVA followed by Tukey's post-hoc test, P < 0.05. LPS lipopolysaccharide, TMZ Trimetazidine, Esc escitalopram, MMP-9 matrix metallopeptidase 9

The LPS administration has a detrimental effect on BBB integrity and function. LPS-injected mice displayed 2.4-folds elevation in the hippocampal content of MMP-9, an important protein involved in maintaining BBB integrity and function (F_(4, 25)_ = 122.4, p < 0.0001). This was associated with dysregulation in BBB tight junctional proteins as exemplified by showing a considerable depression in the hippocampal content of claudin-5 (67%) leading to BBB dysfunction (F_(4, 25)_ = 146.9, p < 0.0001). Such effects were hindered by the TMZ treatment which led to MMP-9 downregulation by 47%, while it enhanced the claudin-5 concentration in the hippocampi by 2.3-folds. These effects were almost analogous to that obtained by LPS-injected mice treated with Esc which reduced MMP-9 by 38%, while elevated claudin-5 level by 2.5-folds, as compared to the LPS group **(**Fig. 8**)**.

## Discussion

The current study investigated the antidepressant effect of TMZ; an anti-ischemic drug used for coronary artery disease, in LPS-induced depressive behaviors in mice. TMZ diminished LPS-associated oxidative stress, neuro-inflammation, and apoptosis in addition to restoration of serotonin levels via down regulation of SERT. The neuroprotective effects exerted by TMZ can be attributed to its modulatory effects on TLR4/NF-κB and Nrf2/HO-1 pathways which enforced BBB integrity and reduced despair behavior recorded by FST.

Treatment with TMZ alleviated LPS–induced animals’ despair witnessed in FST as reflected by significant decrement in the immobility time. Interestingly, TMZ-associated mood enhancement was analogous to the standard antidepressant used; Esc. Regarding the locomotor activity and rearing frequency detected by OFT, there was no significant difference among the experimental groups. Similarly, administration of LPS every other day for 2 weeks was formerly linked to increased immobility time in FST reflecting animals' despair. Meanwhile, LPS injection didn't alter the animals' spontaneous locomotor activity and rearing frequency recorded during OFT (Stupp et al. 2018; Abdel Rasheed et al. 2023). Conversely, injection of LPS was reported to induce a significant decrease in locomotor activity recorded in OFT (Mustapha et al. 2022). This contradiction could be attributed to LPS different dose regimens and mice species used which are linked to LPS-associated locomotor derangements (Yin et al. 2023). Moreover, some evidence stated that LPS-induced decrement in locomotor activity was attributed to the sickness behavior associated with LPS, yet this effect is transient as it soon develops tolerance (Lim et al. 2020a; Kahn et al. 2012).

Administration of TMZ attenuated LPS–induced neuro-inflammation with prominent inhibition of NF-κBp65 leading to an obvious decline of TNF-α and IL-1β levels owing to TLR4 downregulation. TMZ-induced anti-inflammatory effects were equivalent to the reputable antidepressant drug; Esc. Accordingly, LPS-induced stimulation of TLR4 was reported to promote the recruitment of inflammatory cells which is implicated in NF-κBp65 activation with consequent release of the formerly mentioned pro-inflammatory mediators namely; TNF-α and IL-1β (Xu et al. 2020; Qiu et al. 2021).

In the present study, inhibition of TLR4/NF-κB pathway via the administration of TMZ preserved the BBB integrity as it diminished LPS–induced neuro-inflammation leading to a significant decrease in MMP-9 content contrary to claudin-5 enhanced content. In line with the current investigation findings, activation of TLR4/NF-κB signaling is implicated in the marked disruption of BBB permeability associated with LPS injection (Hu et al. 2017). Moreover, it was reported that LPS is implicated in MMP-9 induction via NF-κB leading to claudin-5 degeneration and BBB dysfunction (Huang et al. 2021; Yang et al. 2020).

Administration of TMZ, in the current study, reduced SERT expression and consequently induced an upsurge in serotonin which enforces TMZ antidepressant activity. TMZ–induced SERT downregulation could be attributed to its inhibitory action on TLR4/NF-κB pathway. On the other hand, LPS, probably via its well-known pro-inflammatory effects, is correlated to the observed SERT upregulation with consequent depressive behaviors (Zhu et al. 2015). Moreover, LPS–associated elevation of TNF-α and IL-1β levels owing to TLR4/NF-κB cascade activation was reported to promote rapid activation of SERT (Zhu et al. 2006).

Treatment with TMZ diminished LPS–induced triad of oxidative stress, neuro-inflammation, and apoptosis leading to augmented contents of Nrf2, HO-1, and GSH. These effects are attributed to the prominent inhibition of the culprit inflammatory cascade; TLR4/NF-κB pathway along with MMP-9 and caspase-3 dismounted levels. TMZ neuroprotective effects were equivalent to Esc regarding the formerly mentioned parameters except for Nrf2 content whose elevation in LPS + Esc group out weighted that recorded in LPS + TMZ group. Several studies stated that LPS–induced neuro-inflammation could be attributed principally to its inhibitory action on Nrf2 pathway contrary to NF-κB upregulation which is a key player in BBB dysfunction, neuro-inflammation, and apoptosis (Lim et al. 2020b; Kobayashi et al. 2016; Ren et al. 2019). Besides, Nrf2 has the potential to inhibit LPS–induced up-regulation of pro-inflammatory cytokines as well as MMP-9 which induces BBB disruption and cell death (Kobayashi et al. 2016; Mao et al. 2010). Nrf2 is also Known to regulate the expression of various antioxidant and anti-inflammatory mediators such as HO-1 in addition to the antioxidant enzymes involved in GSH synthesis (Ahmad et al. 2023).

Finally, in the present investigation, the TMZ administration attenuated LPS–induced microglial activation and neuronal damage, an effect that supports its neuroprotective effects against LPS–induced neurodegeneration owing to extravagant neuroinflammation. LPS has been formerly linked to microglial activation with prominent decline in neuronal survival especially in the hippocampal brain region (M. A., Jaya, M. Hayashida, K. Tsuchie, S. J. F., Jerin, R. Mamunur, S. Miura M. Inajaki 2022; Jung et al. 2023). Moreover, LPS has been reported to promote glial activation via the upregulation of TLR4/NF-κB signaling leading to massive release of pro-inflammatory cytokines namely; TNF-α and IL-1β (Zhao et al. 2019). In line with the present study findings, TMZ was reported to exert anti-inflammatory and cytoprotective roles in various body tissues, especially the nervous tissue (Atilgan et al. 2014).

Based on the present work results, TMZ is considered as a promising antidepressant which provides new insights into depression pathogenesis and encourages adopting novel therapeutic strategies with noticeable efficacy and fewer drawbacks than those recorded with the traditional antidepressants.

## Conclusion

TMZ; a well-known anti-ischemic drug, exerted mood boosting effects in LPS-injected mice. Moreover, it was able to overcome LPS-induced vicious cycle of oxidative stress, neuro-inflammation, and apoptosis probably via its ability to fine tune NF-κBp65 and Nrf2 correlation and replenish depression-associated serotonin deficiency. TMZ also preserved BBB integrity and opposed LPS-associated despair behavior witnessed in FST. Consequently, TMZ is a prosperous candidate that needs further investigations to authenticate its antidepressant activity. Meanwhile, TMZ neuroprotective effects should be investigated in different animals, for example rats, and in various depression models to explore its antidepressant activity in several milieus in order to establish a reasonable assessment of its antidepressant potential. Finally, extrapolation of the current investigation findings to humans is mandatory to validate TMZ favorable effects in humans with emphasis on various depression aspects besides the neuro-inflammatory milieu as anxiety and cognitive dysfunction associated with the disease pathogenesis.

## Limitations

Extrapolation of the present study findings to humans is necessary to confirm TMZ antidepressant effects in humans with emphasis on various depression aspects besides the neuro-inflammatory milieu. Moreover, depression is a complex disorder with plenty of symptoms such as lack of interest, anxiety, and cognitive decline which are not easily mimicked by animal models of depression. Therefore, studying TMZ antidepressant activity in humans will be a great merit.

## Data Availability

No datasets were generated or analysed during the current study.
